# A cross-sectional analysis of the relationship between uric acid and coronary atherosclerosis in patients with suspected coronary artery disease in China

**DOI:** 10.1186/1471-2261-14-101

**Published:** 2014-08-16

**Authors:** Yujiao Sun, Xin Yu, Ying Zhi, Song Geng, Hua Li, Ting Liu, Ke Xu, Ling Chen, Chunwei Wu, Guoxian Qi

**Affiliations:** 1 Department of Cardiology of Aging, Department of Cardiology, The First Affiliated Hospital of China Medical University, NO.155 Nanjing North Street, Heping Ward, Shenyang 110001, China; 2 Department of Radiology, The First Affiliated Hospital of China Medical University, Shenyang, China

**Keywords:** Uric acid, Coronary atherosclerosis, Coronary computed tomography angiography, Gender, Calcium score

## Abstract

**Background:**

Although many studies have examined the relationship between uric acid (UA) and coronary artery disease (CAD), whether UA is an independent risk factor contributing to progression of CAD is still controversial. Whether UA plays a different role in different sexes is also unclear.

**Methods:**

A total of 1116 individuals with suspected CAD were stratified into four groups according to their serum UA quartiles in total (men and women combined), in men, and in women. The association of UA with coronary atherosclerosis was assessed by univariable and multivariable logistic regression.

**Results:**

In total and in women, the prevalence of any plaques and significant/severe stenosis was significantly increased with an increase in quartiles of UA (all P < 0.05). The proportion of triple-vessel disease and left main artery lesion was highest in the fourth quartile (both p < 0.05). Increasing quartiles of UA were significantly associated with a coronary artery calcium score (CACS) >10 (all P < 0.01). As UA levels increased in women, the incidence of double-vessel lesions (p = 0.017) and the proportion of mixed plaques (p = 0.022) were significantly increased. The proportion of a CACS of 0 in total, in men and women was highest in the first quartile (all P < 0.01). UA was the strongest predictor of significant stenosis, multivessel disease, and mixed plaques in women (all p < 0.05). UA was the only risk factor for mixed plaques in total (P = 0.046).

**Conclusion:**

The level of UA was significantly associated with coronary atherosclerosis in women, but not men.

## Background

Uric acid (UA) is the main end product of purine catabolism [1]. High UA levels are often accompanied by obesity, hyperlipidemia, hypertension, glucose intolerance, and insulin resistance [2-5], which contribute to the development of cardiovascular disease. Elevated UA levels are associated with coronary artery disease (CAD), independently of traditional CAD risk factors [6,7]. However, some studies have suggested that UA is only considered as a risk marker or an adaptive ascended to attempt to prevent atherosclerosis [8-10], and this may be due to its antioxidant properties [11]. Although studies have examined the relationship between UA and CAD, whether increased UA is an independent risk factor that contributes to early CAD is still controversial.

Sex might be an important factor involved in the relationship between UA and CAD. In a subgroup analysis of LIFE [12], the relationship between UA and CAD was significant only in women. A meta-analysis [6] found that UA was significantly correlated with CAD only in women. A strong association between UA and cardiac events has been observed in both sexes in other studies [13,14]. Whether UA plays a different role in different sexes in the progression of CAD is unclear.

Therefore, in this study, we assessed the association between UA and coronary atherosclerosis in patients with suspected CAD who underwent 256-detector-row coronary computed tomographic angiography (CCTA). We further assessed these associations in sex subgroups.

## Methods

### Study population

**Figure 1:**
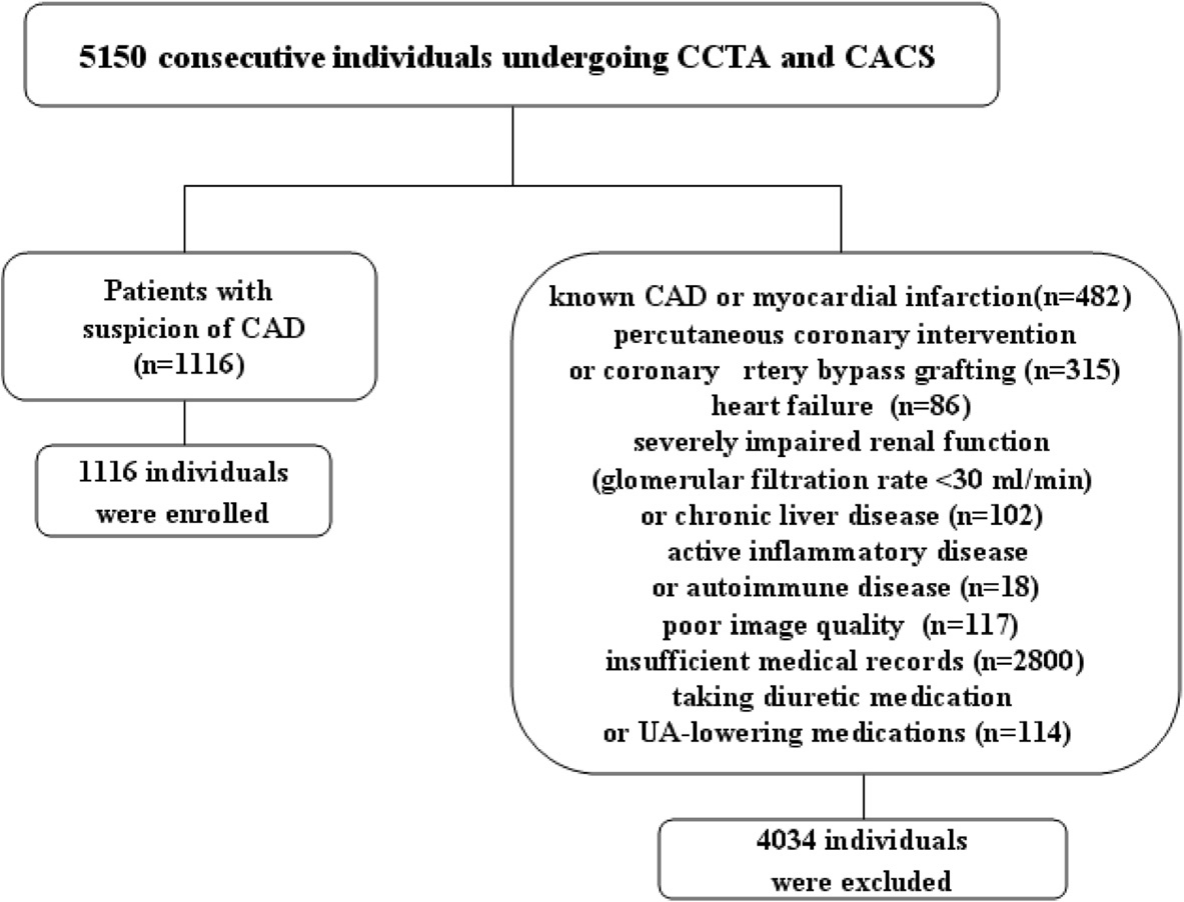
The path of patients enrollment.

This study included 5150 consecutive individuals (≥18 years) in China undergoing CCTA and coronary artery calcium score (CACS) measurements in our institution from September 2011 to February 2013. CCTA and measurement of the CACS were performed for the suspicion of CAD after clinical assessment (including cardiac symptoms, risk factors, electrocardiogram changes, and a positive stress test). Finally, 1116 individuals were enrolled (Figure 1). All patients gave written inform consent, and the study was approved by the ethics committee of the First Affiliated Hospital of China Medical University.

### Assessment of CAD risk factors

All patients were systematically asked about their demographics by professionals. Body weight, height, and blood pressure were measured. Hypertension was defined as a previously established diagnosis and/or antihypertensive medication, systolic blood pressure ≥140 mmHg, and diastolic blood pressure ≥90 mmHg. Diabetes mellitus was defined as a previously established diagnosis and/or antidiabetic treatment, and fasting glucose ≥126 mg/dl. A family history of CAD was defined as a first-degree male relative aged <55 years or a first-degree female relative aged <65 years. Smoking was defined as any cigarette smoking within 1 year of CCTA. Medication use was recorded in detail.

Total cholesterol (TC), triglycerides (TG), high-density lipoprotein cholesterol (HDL-C), low-density lipoprotein cholesterol (LDL-C), creatinine and UA levels were measured after at least a 12-h fasting period within 7 days of CCTA. The contents of UA were measured with enzyme kinetics in all enrolled patients, and it was uniform throughout the study period.

### Acquisition of images

Computed tomography scans were performed using 256-detector-row CCTA (Brilliance; Philips Medical System, The Netherlands). Individuals with a heart rate ≥75 beats per minute were treated orally with up to 100 mg metoprolol for several hours (except for contraindications of beta-blockers) before CCTA imaging to achieve a higher image quality. First, after a scout radiograph of the chest (lateral and anteroposterior), a noncontrast CACS scan was performed, and the image section thickness was 2.5 mm by triggering at a heart rate depending on the percentage of the R-R interval. The sections were collected from the level of the carina and proceeded to the level of the diaphragm. Thereafter, retrospective electrocardiogram-gated contrast-enhanced CCTA was performed. The CCTA scan was initiated 20 mm above the level of the left main artery to 20 mm below the inferior myocardial apex during a single breath-hold. Depending on the individual’s weight, a bolus of 50–80 ml of iopamidol or iohexol was intravenously injected at 4–5.5 ml/s into the antecubital vein, followed by 50 ml of saline. A standard scan protocol was applied, with section collimation of 256 × 0.625 mm, 0.27 s for rotation time, 120 KV tube voltage, and 800–1100 mA tube current. In all scans, electrocardiogram-gate dose modulation was used. The electrocardiograms of individuals were simultaneously collected to allow for retrospective segmental data reconstruction. The images of all individuals were initially reconstructed at 75% of the R-R interval of the cardiac cycle. If motion artefacts were found, reconstruction of additional phases was performed. The best R-R interval image quality was chosen for interpretation. The dose range of radiation for CCTA was estimated to be 10–18 mSv.

### Image analysis

All images were analysed separately by two experienced radiologists and one cardiologist who were blinded to the patients’ characteristics. Consensus on interpretation was performed to achieve a final CCTA diagnosis. All scans were evaluated by a three-dimensional workstation (Brilliance; Philips Medical Systems). The CACS was measured using the scoring system previously described by Agatston et al. [15].

The three reads were permitted to use any/all available post-processing image reconstruction algorithms, including two-dimensional axial or three-dimensional maximal intensity projection, multiplanar reformat, cross-sectional analysis, or the volume-rendered technique. A 16-segment coronary artery tree model [16] was used in the analysis of coronary arteries. In each coronary segment, plaques were defined as any tissue structure >1 mm^2^, which existed either within the coronary artery lumen or was adjacent to the coronary artery lumen, and could be discriminated from surrounding pericardial tissue, epicardial fat, or the vessel lumen itself. For evaluating the degree of stenosis, the coronary lumen was semi-automatically traced at the maximal stenosis site and was compared with the mean value of a proximal and distal reference site. The image quality was evaluated and classified as follows: good, with no artifact; adequate, with the presence of artifacts but feasible for evaluating the degree of stenosis and plaque characteristics; or poor, with the presence of artifacts and not feasible for evaluating the degree of stenosis and plaque characteristics. If an image was graded as poor, the image was not included.

Coronary lesions ≥50 and 70% were defined as significant and severe, respectively. Multivessel disease was defined as the presence of stenosis of more than 50% in at least two vessels. The stenosed coronary vessel of individuals was further categorized as having one-, two-, and three-vessel/left main disease. All detected plaques were classified as calcified, non-calcified, or mixed. The calcified component of a stenosis was defined as a lesion with radiodensity greater than the luminal contrast. The non-calcified component of a stenosis was defined as a lesion with radiodensity greater than that of neighbouring soft tissue and lower than the luminal contrast. Plaques that contained calcified tissue greater than 75% of the plaque area were classified as calcified plaques, less than 25% as non-calcified plaques, and 25–75% as mixed plaques [17].

### Statistical analysis

Baseline characteristics are expressed as absolute counts and proportions for categorical variables, and as means ± standard deviations for continuous variables. Continuous variables were analysed by analysis of variance and categorical variables were analysed by *χ*^2^ test.

The association of UA with coronary atherosclerosis was assessed by the *χ*^2^ test. Univariable and multivariable logistic regression were used to assess the association of UA with significant stenosis, multivessel disease, high CACS, and plaque characteristics. Variables that showed a P value <0.1 with univariable analysis were applied to multivariate analysis. All analyses were performed using SPSS 15.0. The level of significance was set at *P* < 0.05.

## Results

### Baseline characteristic baseline characteristics

**Figure 2:**
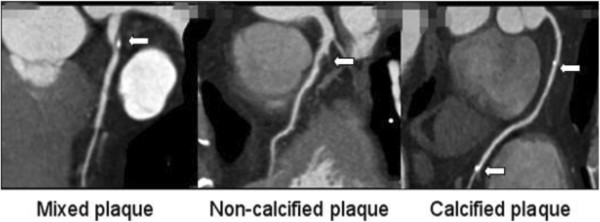
Showed the representative view of mixed, non-calcified plaque.

Among the 1116 patients in the study, 50.7% were men, and the mean age was 58.05 ± 10.69 years. All individuals were stratified into four groups according to their UA quartile, the first quartile:<259 μmmol/l (n = 278); the second quartile: 259–309 μmmol/l (n = 274); the third quartile: 310-373 μmmol/l (n = 283) and the fourth quartile: >373 μmmol/l (n = 281). With increasing UA quartiles, the prevalence of male sex, smoking, and hypertension was significantly increased (all *P* < 0.01). Body mass index (BMI) and TC level were significantly higher (all *P* < 0.001) and HDL-C level was significantly lower (*P* < 0.001, Table 1). Figure 2 showed the representative view of mixed, non-calcified and calcified plaque.

**Table 1:** Baseline characteristics of the all study population according to the quartiles of the serum uric acid

**Variables**	**Total (n = 1116)**	**1st Quartile <259 μmol/l (n = 278)**	**2nd Quartile 259-309 μmol/l (n = 274)**	**3rd Quartile 310-373 μmol/l (n = 283)**	**4th Quartile >373 μmol/l (n = 281)**	**P-value**
Age(years)	58.05 ± 10.69	58.64 ± 9.19	57.86 ± 9.52	58.14 ± 11.09	57.56 ± 12.6	0.674
Male	566 (50.7)	64 (23.0)	109 (39.8)	170 (60.1)	223 (79.4)	<0.001
BMI(kg/m^2^)	24.99 ± 4.00	23.46 ± 4.24	24.70 ± 3.29	25.83 ± 4.45	25.95 ± 3.38	<0.001
Smoking	279 (25.0)	40 (14.4)	59 (21.5)	89 (31.4)	91 (32.4)	<0.001
Family history of CAD	93 (8.3)	27 (9.7)	29 (10.6)	20 (7.1)	17 (6.0)	0.167
Hypertension	639 (42.7)	96 (34.5)	114 (41.6)	135 (47.7)	132 (47.0)	0.005
Diabetes Mellitus	183 (16.4)	45 (16.2)	43 (15.7)	51 (18.0)	44 (15.7)	0.861
LDL-C(mmol/l)	2.95 ± 0.88	2.93 ± 0.79	3.02 ± 1.01	2.94 ± 0.87	2.92 ± 0.87	0.544
HDL-C(mmol/l)	1.19 ± 0.37	1.33 ± 0.34	1.21 ± 0.32	1.13 ± 0.30	1.09 ± 0.46	<0.001
TC(mmol/l)	4.60 ± 1.06	4.61 ± 0.97	4.66 ± 1.22	4.58 ± 1.06	4.56 ± 0.99	0.735
TG(mmol/l)	1.64 ± 1.36	1.29 ± 0.85	1.59 ± 1.29	1.69 ± 1.47	1.98 ± 1.61	<0.001
Creatinine(μmol/l)	78.20 ± 16.32	81.47 ± 16.74	80.09 ± 14.23	74.07 ± 15.38	81.95 ± 19.17	0.121
History of Medication						
Aspirin	247 (22.1)	64 (23.0)	57 (20.8)	67 (23.7)	59 (21.0)	0.799
Beta blcker	118 (10.6)	27 (9.7)	26 (9.5)	31 (11.0)	34 (12.1)	0.733
ACEI	62 (5.6)	13 (4.7)	15 (5.5)	17 (6.0)	17 (5.6)	0.880
ARB	68 (6.1)	14 (5.0)	13 (4.7)	20 (7.1)	21 (7.5)	0.417
CCB	145 (13.0)	28 (10.1)	43 (15.7)	42 (14.8)	32 (11.4)	0.144
Statins	94 (8.4)	20 (7.2)	25 (9.1)	30 (10.6)	19 (6.8)	0.330
Antihyperglycemic	107 (9.6)	30 (10.8)	23 (8.4)	29 (10.2)	25 (8.9)	0.750
Insulin	65 (5.8)	18 (6.5)	14 (5.1)	18 (6.4)	15 (5.3)	0.863

Values are mean ± Standard deviation or n (%). The conversion of UA measuring unit: mg/dl = (μmol/l divide by 59.48).

*BMI* Body Mass Index, *CAD* Coronary artery disease, *LDL-C* Low-density lipoprotein Cholesterol, *HDL-C* High-density lipoprotein Cholesterol, *TC* Total Cholesterol, *TG* Triglycerides, *ACEI* Angiotensin-converting enzyme inhibitor, *ARB* Angiotensin receptor blocker, *CCB* Calcium Channel Blocker.

### Prevalence, characteristics, and the CACS of coronary artery plaques

**Figure 3:**
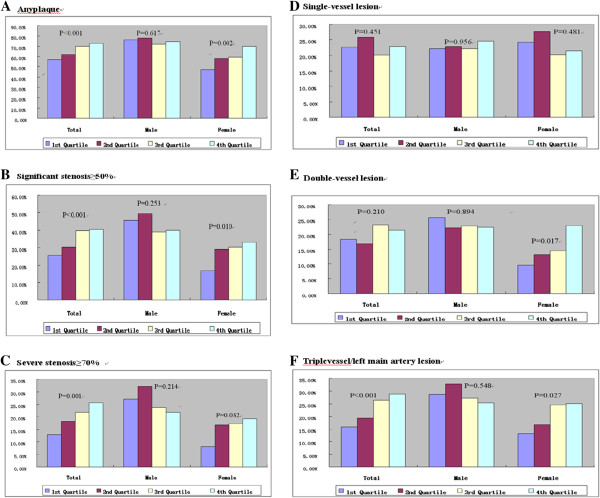
Relationship with anyplaque (A), significant stenosis (B) and severe stenosis (C), single vessel lesion (D), double vessel lesion (E) and triple vessel/left main artery lesion (F) across quartile of serum uric acid levels across quartile of serum uric acid levels.

Men and women were stratified into four groups according to their UA quartile. The quartiles of UA in men were the first quartile: 296 μmmol/l (n = 140); the second quartile: <296-349 μmmol/l (n = 140); the third quartile: 350-406 μmmol/(n = 144) and the fourth quartile: >406 μmmol/l (n = 142). The quartiles of UA in women were the first quartile: <238 μmmol/l (n = 136); the second quartile: 238-273 μmmol/l (n = 137); the third quartile: 274-326 μmmol/l(n = 138) and the fourth quartile > 326 μmmol/l (n = 139). With increasing UA quartiles in total (men and women combined) and in women, the prevalence of plaques were significantly increased (total: 56.8% vs 62.0% vs 70.0% vs 73.0%, *P* < 0.001; women: 47.1% vs 57.7% vs 59.4% vs 69.8%, p = 0.002, (Figure 3A), significant stenosis were significantly increased (total: 25.5% vs 30.3% vs 39.6% vs 40.2%, *P* < 0.001; women: 16.9% vs 29.2% vs 30.4% vs 33.1%, *P* = 0.010, Figure 3B), and severe stenosis were significantly increased (total: 12.9% vs 18.2% vs 21.9% vs 25.6%, *P* = 0.001; women: 8.1% vs 16.8% vs 17.4% vs 19.4%, *P* = 0.032, Figure 3C).

The incidence of double-vessel lesions was only significantly increased with UA quartiles in women (9.6% vs 13.1% vs 14.5% vs 23.0%, *P* = 0.017, Figure 3E). In total and in women, the proportion of triple-vessel/left main artery lesions were highest in the fourth quartile (total: 15.8% vs 19.3% vs 26.5% vs 28.8%, *P* < 0.001; women: 13.2% vs 16.8% vs 24.6% vs 25.2%, *P* = 0.027, Figure 3F).

**Figure 4:**
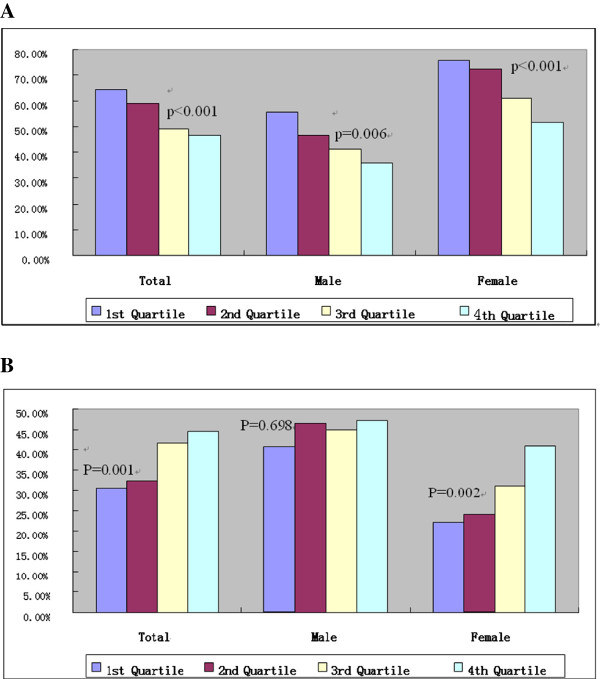
Prevalence of coronary artery calcium score = 0 across quartile of serum uric acid levels (A), prevalence of coronary artery calcium score >10 across quartile of serum uric acid levels (B).

**Figure 5:**
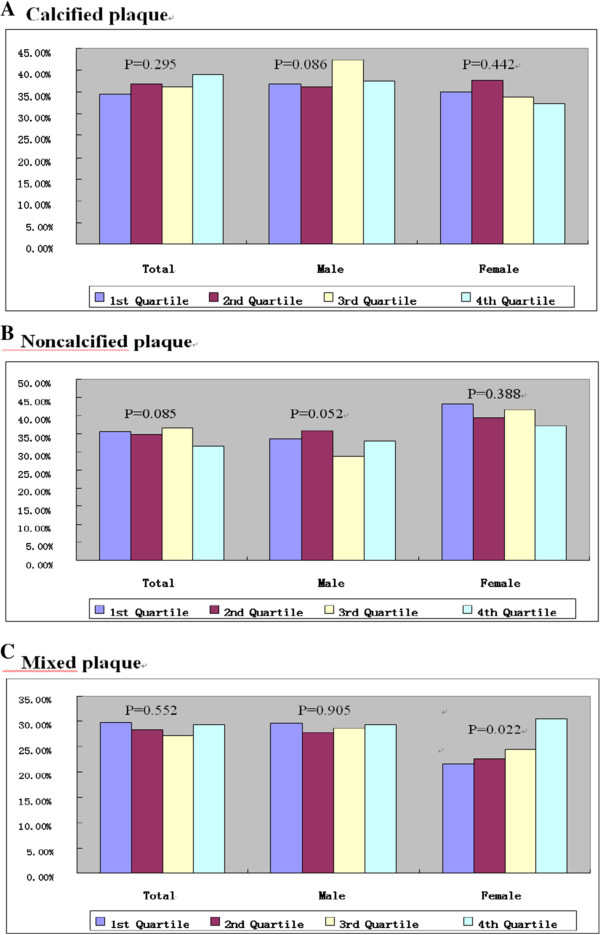
Relationship with (A) calcified, noncalcified (B) and mixed (C) across quartile of serum uric acid levels.

The proportion of a CACS of 0 in total, in men, and in women were lowest in the fourth quartile (total: 64.4% vs 59.1% vs 49.1% vs 46.6%, *P* < 0.001; men: 55.7% vs 46.4% vs 41.0% vs 35.9%, *P* = 0.006; women: 75.7% vs 72.3% vs 60.9% vs 51.8%, *P* < 0.001, Figure 4A). The increasing quartiles of UA were significantly associated with a CACS >10 in total (30.6% vs 32.5% vs 41.7% vs 44.5%, *P* = 0.001) and in women (22.1% vs 24.1% vs 31.2% vs 41.0%, p = 0.002, Figure 4B.). As UA levels increased in women, the proportion of mixed plaques significantly increased (21.7% vs 22.7% vs 24.5% vs 30.5%, *P* = 0.022, Figure 5C).

### Univariate and multivariate logistic regression models

In multivariate analysis for men and women combined, age, male sex, smoking, hypertension, and diabetes mellitus (DM) were significantly associated with significant stenosis, multivessel disease, and a high CACS (all p < 0.001). HDL-C level was only significantly associated with significant stenosis and multivessel disease (both *P* ≤ 0.002). UA was the strongest predictor for significant stenosis, multivessel disease, and a high CACS in univariate analysis (all *P* < 0.001), but this association was not apparent after adjustment (Table 2).

**Table 2:** Univariate and multivariate logistic regression models for variables of total population associated with coronary artery lesions and high CACS (n = 1116)

**V variable**	**Significant stenosis (>50%)**				**Multivessel disease**				**High CACS (CACS > 100)**			
	**Univariate**		**Multivariate**		**Univariate**		**Multivariate**		**Univariate**		**Multivariate**	
**Variable**	**OR (95%CI)**	**P**	**OR (95%CI)**	**P**	**OR (95%CI)**	**P**	**OR (95%CI)**	**P**	**OR (95%CI)**	**P**	**OR (95%CI)**	**P**
**Age**	1.059 (1.046-1.073)	<0.001	1.070 (1.054-1.086)	<0.001	1.066 (1.052-1.081)	<0.001	1.079 (1.062-1.096)	<0. 001	1.078 (1.061-1.095)	<0.001	1.089 (1.070-1.108)	<0.001
**Male**	2.364 (1.830-3.054)	<0.001	2.208 (1.579-3.089)	<0.001	2.365 (1.815-3.083)	<0.001	2.320 (1.637-3.288)	<0.001	2.244 (1.653-3.047)	<0.001	2.190 (1.486-3.226)	<0.001
**BMI**	1.028 (0.997-1.060)	0.082	1.010 (0.975-1.047)	0.568	1.027 (0.996-1.060)	0.092	1.010. (0.974-1.047)	0.597	1.017 (0.982-1.052)	0.345	----------	-----
**Smoking**	1.789 (1.355-2.364)	<0.001	1.835 (1.306-2.578)	<0.001	1.739 (1.308-2.310)	<0.001	1.866 (1.313-2.653)	0.001	1.854 (1.352-2.542)	<0.001	2.294 (1.550-3.395)	<0.001
**Family history of CAD**	1.076 (0.690-1.678)	0.746	----------	-----	1.105 (0.701-1.742)	0.666	----------	-----	1.012 (0.598-1.714)	0.964	----------	-----
**Hypertension**	2.187 (1.700-2.815)	<0.001	1.856 (1.394-2.471)	<0.001	2.275 (1.754-2.950)	<0.001	1.914 (1.421-2.577)	<0.001	2.377 (1.764-3.203)	<0.001	1.939 (1.388-2.709)	<0.001
**DM**	2.467 (1.788-3.403)	<0.001	1.825 (1.278-2.604)	<0.001	2.823 (2.041-3.905)	<0.001	2.153 (1.501-3.089)	<0.001	2.403 (1.694-3.408)	<0.001	1.983 (1.344-2.927)	0.001
**LDL-C**	0.876 (0.758-1.012)	0.072	1.156 (0.812-1.645)	0.421	0.913 (0.787-1.059)	0.228	----------	-----	1.061 (0.900-1.251)	0.482	----------	-----
**HDL-C**	0.331 (0.202-0.477)	<0.001	0.456 (0.276-0.755)	0.002	0.311 (0.202-0.477)	<0.001	0.428 (0.256-0.717)	0.001	0.632 (0.396-1.008)	0.054	0.907 (0.559-1.471)	0.691
**TC**	1.802 (1.724-1.929)	0.002	0.875 (0.641-1.194)	0.399	1.845 (1.744-1.959)	0.009	1.022 (0.885-1.181)	0.765	0.943 (0.819-1.086)	0.415		
**TG**	0.983 (0.895-1.081)	0.726	----------	-----	0.992 (0.901-1.092)	0.863	----------	-----	0.889 (0.771-1.024)	0.145	----------	-----
**UA**	1.003 (1.002-1.004)	<0.001	1.001 (0.999-1.002)	0.535	1.003 (1.002-1.004)	<0.001	1.001 (0.999-1.002)	0.564	1.003 (1.001-1.005)	<0.001	1.001 (0.999-1.003)	0.205
**Creatinine**	0.980 (0.857-1.018)	0.120	----------	-----	1.240 (0.957-1.417)	0.258	----------	-----	0.760 (0.526-1.013)	0.173	----------	-----

Multivessel disease was defined as the present of stenosis of more than 50% in at lease two vessels.

Risk factors with p < 0.10 in unvariate adjusted simultaneously. Blanks indicate variables not adjusted in multivariate analyses.

The same analyses were performed for men and women (Tables 3 and 4). For men, significant predictors of significant stenosis were age, hypertension, and HDL-C levels after adjustment (all p < 0.05). Significant predictors of multivessel disease were age and hypertension after adjustment (both *P* < 0.05). The strongest risk factors for a high CACS were age and hypertension after adjustment (both *P* < 0.05, Table 3). For women, significant predictors of stenosis were age, hypertension, DM, and UA after adjustment (all *P* < 0.05). Significant predictors of multivessel disease were age, hypertension, DM, and UA after adjustment (all *P* < 0.05). The strongest risk factors for a high CACS were age, hypertension, DM, and HDL-C levels after adjustment (all *P* < 0.05, Table 4).

**Table 3:** Univariate and multivariate logistic regression models for variables of male associated with coronary artery lesions and high CACS (n = 566)

**V variable**	**Significant stenosis (>50%)**				**Multivessel disease**				**High CACS (CACS > 100)**			
	**Univariate**		**Multivariate**		**Univariate**		**Multivariate**		**Univariate**		**Multivariate**	
**Variable**	**OR (95%CI)**	**P**	**OR (95%CI)**	**P**	**OR (95%CI)**	**P**	**OR (95%CI)**	**P**	**OR (95%CI)**	**P**	**OR(95%CI)**	**P**
**Age**	1.056 (1.039-1.073)	<0.001	1.052 (1.034-1.070)	<0.001	1.060 (1.043-1.077)	<0.001	1.055 (1.037-1.073)	<0.001	1.077 (1.057-1.098)	<0.001	1.071 (1.050-1.093)	<0.001
**BMI**	0.978 (0.937)1.020	0.296	----------	-----	0.979 (0.938-1.022)	0.329	----------	-----	0.955 (0.906-1.006)	0.084	0.996 (0.945-1.051)	0.894
**Smoking**	1.245 (0.889-1.743)	0.203	----------	-----	1.298 (0.926-1.820)	0.131	----------	-----	1.305 (0.896-1.900)	0.166	----------	-----
**Family history of CAD**	1.274 (0.684-2.375)	0.446	----------	-----	1.501 (0.805-2.798)	0.201	----------	-----	1.080 (0.539-2.162)	0.828	----------	-----
**Hypertension**	1.811 (1.291-2.541)	0.001	1.579 (1.098-2.270)	0.014	1.734 (1.235-2.434)	0.001	1.462 (1.016-2.104)	0.041	2.044 (1.400-2.983)	<0.001	1.731 (1.138-2.634)	0.010
**DM**	1.608 (1.045-2.473)	0.031	1.368 (0.862-2.172)	0.184	1.727 (1.123-2.658)	0.013	1.579 (0.998-2.499)	0.051	1.585 (1.001-2.511)	0.049	1.581 (0.952-2.624)	0.077
**LDL-C**	0.856 (0.694-1.054)	0.143	----------	-----	0.919 (0.746-1.133)	0.430	----------	-----	0.986 (0.782-1.243)	0.902	----------	-----
**HDL-C**	0.575 (0.317-1.044)	0.069	0.485 (0.236-0.995)	0.048	0.635 (0.350-1.152)	0.135	----------	-----	0.435 (0.072-0.797)	0.030	1.676 (0.813-3.453)	0.162
**TC**	1.764 (1.641)1.911	0.003	0.880 (0.712-1.088)	0.239	1.817 (1.688-1.971)	0.022	0.875 (0.721-1.062)	0.176	0.891 (0.738-1.075)	0.229	----------	-----
**TG**	1.868 (1.759-1.994)	0.041	0.939 (0.797-1.106)	0.451	0.874 (0.763-1.001)	0.051	0.998 (0.864-1.152)	0.973	1.754 (1.616-1.924)	0.006	0.926 (0.754-1.137)	0.464
**UA**	1.000 (0.998-1.002)	0.923	----------	-----	1.000 (0.998-1.002)	0.716	----------	-----	1.000 (0.998-1.002)	0.960	----------	-----
**Creatinine**	1.403 (0.549-3.073)	0.613	----------	-----	0.923 (0.238-1.997)	0.588	----------	-----	0.574 (0.185-1.236)	0.339	----------	-----

Multivessel disease was defined as the present of stenosis of more than 50% in at lease two vessels.

Risk factors with p < 0.10 in unvariate adjusted simultaneously. Blanks indicate variables not adjusted in multivariate analyses.

**Table 4:** Univariate and multivariate logistic regression models for variables of female associated with coronary artery lesions and high CACS (n = 550)

**V variable**	**Significant stenosis (>50%)**				**Multivessel disease**				**High CACS (CACS > 100)**			
	**Univariate**		**Multivariate**		**Univariate**		**Multivariate**		**Univariate**		**Multivariate**	
**Variable**	**OR (95%CI)**	**P**	**OR (95%CI)**	**P**	**OR (95%CI)**	**P**	**OR (95%CI)**	**P**	**OR (95%CI)**	**P**	**OR (95%CI)**	**P**
**Age**	1.096 (1.069-1.123)	<0.001	1.084 (1.055-1.113)	<0.001	1.092 (1.068-1.117)	<0.001	1.075 (1.049-1.101)	<0.001	1.100 (1.068-1.133)	<0.001	1.085 (1.049-1.122)	<0.001
**BMI**	1.071 (1.013-1.132)	0.015	1.024 (0.985-1.103)	0.151	1.073 (1.018-1.132)	0.009	1.036 (0.980-1.095)	0.216	1.077 (1.012-1.147)	0.020	1.060 (0.998-1.126)	0.060
**Smoking**	1.434 (0.723-2.845)	0.303	----------	-----	1.317 (0.692-2.504)	0.402	----------	-----	1.795 (0.822-3.918)	0.142	----------	-----
**Family history of CAD**	0.978 (0.496-1.932)	0.950	----------	-----	0.716 (0.376-1.363)	0.309	----------	-----	1.017 (0.440-2.351)	0.969	----------	-----
**Hypertension**	3.144 (2.096-4.718)	<0.001	2.229 (1.420-3.500)	<0.001	3.689 (2.550-5.336)	<0.001	2.618 (1.735-3.950)	<0.001	3.385 (2.017-5.680)	<0.001	2.162 (1.216-3.843)	0.009
**DM**	4.144 (2.536-6.772)	<0.001	2.669 (1.554-4.584)	<0.001	4.301 (2.620-7.061)	<0.001	2.913 (1.682-5.044)	<0.001	4.286 (2.476-7.418)	<0.001	2.958 (1.582-5.531)	0.001
**LDL-C**	1.049 (0.851-1.293)	0.652	----------	-----	1.044 (0.863-1.263)	0.654	----------	-----	1.381 (1.078-1.769)	0.011	2.090 (0.868-5.036)	0.100
**HDL-C**	0.309 (0.158-0.602)	<0.001	0.462 (0.219-0.977)	0.430	0.344 (0.192-0.618)	<0.001	0.642 (0.336-1.227)	0.180	0.265 (0.112-0.629)	0.003	0.327 (0.118-0.908)	0.032
**TC**	1.056 (0.878-1.271)	0.561	----------	-----	1.054 (0.890-1.247)	0.544	----------	-----	1.236 (0.991-1.541)	0.060	0.776 (0.346-1.741)	0.776
**TG**	1.190 (0.994-1.424)	0.058	1.043 (0.852-1.276)	0.684	1.338 (1.103-1.623)	0.003	1.131 (0.913-1.401)	0.259	1.094 (0.897-1.334)	0.374	----------	-----
**UA**	1.214 (1.101-1.407)	0.002	1.004 (1.001-1.007)	0.011	1.115 (1.103-1.134)	<0.001	11.131(0.913-1.401) 1.003(1.000-1.006)	<0.001	1.005 (1.001-1.008)	0.004	1.001 (0.997-1.004)	0.793
**Creatinine**	1.835 (0.511-3.164)	0.535	----------	-----	0.714 (0.138-2.227)	0.407	----------	-----	0.925 (0.238-2.764)	0.868	----------	-----

Multivessel disease was defined as the present of stenosis of more than 50% in at lease two vessels.

Risk factors with p < 0.10 in unvariate adjusted simultaneously. Blanks indicate variables not adjusted in multivariate analyses.

In total, significant predictors of significant stenosis were age, male sex, smoking, hypertension, and DM after adjustment (all *P* < 0.05). Significant predictors of non-calcified plaques were age, smoking, hypertension, DM, and HDL-C levels after adjustment (all *P* < 0.05). After adjustment, the strongest risk factors for mixed plaques were age, male sex, smoking, hypertension, DM, and UA (all *P* < 0.05, Table 5). For men, significant predictors of calcified plaques were age, hypertension, DM, and HDL-C after adjustment (all *P* < 0.05). The strongest risk factors for non-calcified plaques were age, smoking, DM, and HDL-C in univariate and multivariate analysis (all *P* < 0.05). The strongest risk factors for a high CACS were age, smoking, and HDL-C levels after adjustment (all *P* < 0.05, Table 6). For women, significant predictors of calcified plaques were age, hypertension, and DM after adjustment (all *P* < 0.05). Significant predictors of non-calcified plaques were age, hypertension, and HDL-C after adjustment (all *P* < 0.05). Age, hypertension, DM, and UA were significantly associated with mixed plaques in univariate and multivariate analysis (all *P* < 0.05, Table 7).

**Table 5:** Univariate and multivariate logistic regression models for variables of total population associated with calcified, non-calcified and mixed plaque (n = 1116)

**V variable**	**Calcified plaque**				**Non-calcified plaque**				**Mixed plaque**			
	**Univariate**		**Multivariate**		**Univariate**		**Multivariate**		**Univariate**		**Multivariate**	
**Variable**	**OR (95%CI)**	**P**	**OR (95%CI)**	**P**	**OR (95%CI)**	**P**	**OR (95%CI)**	**P**	**OR (95%CI)**	**P**	**OR (95%CI)**	**P**
**Age**	1.071 (1.057-1.086)	<0.001	1.052 (1.023-1.081)	<0.001	1.028 (1.016-1.040)	<0.001	1.034 (1.021-1.047)	<0.001	1.056 (1.042-1.069)	<0.001	1.066 (1.051-1.081)	<0.001
**Male**	2.022 (1.579-2.590)	<0.001	2.282 (1.662-3.132)	<0.001	1.666 (1.315-2.111)	<0.001	1.249 (0.925-1.686)	<0.147	2.328 (1.806-3.000)	<0.001	2.002 (1.445-2.774)	<0.001
**BMI**	1.017 (0.987-1.048)	0.276	----------	-----	1.045 (1.012-1.080)	0.008	1.023 (0.988-1.058)	0.202	1.021 (0.991-1.053)	0.173	----------	-----
**Smoking**	1.428 (1.084-1.881)	0.011	1.224 (1.036-1.467)	0.043	1.707 (1.297-2.246)	<0.001	1.729 (1.258-2.376)	0.001	2.171 (1.645-2.864)	<0.001	2.336 (1.669-3.270)	<0.001
**Family history of CAD**	0.825 (0.527-1.293)	0.402	----------	-----	1.183 (0.773-1.810)	0.438	----------	-----	1.022 (0.655-1.592)	0.925	----------	-----
**Hypertension**	2.180 (1.703-2.792)	<0.001	1.832 (1.393-2.409)	<0.001	1.579 (1.244-2.005)	<0.001	1.319 (1.017-1.711)	0.037	2.090 (1.627-2.683)	<0.001	1.810 (1.367-2.395)	<0.001
**DM**	2.538 (1.839-3.503)	<0.001	2.065 (1.524-2.265)	<0.001	1.924 (1.390-2.665)	<0.001	1.510 (1.070-2.130)	0.019	2.386 (1.731-3.291)	<0.001	1.917 (1.346-2.730)	<0.001
**LDL-C**	1.669 (0.930-1.227)	0.348	----------	-----	0.945 (0.826-1.082)	0.945	----------	-----	0.981 (0.852-1.129)	0.786	----------	-----
**HDL-C**	0.851 (0.601-1.206)	0.365	----------	-----	0.347 (0.235-0.512)	<0.001	0.493 (0.322-0.757)	0.001	0.504 (0.338-0.752)	0.001	0.763 (0.501-1.164)	0.210
**TC**	1.009 (0.899-1.132)	0.878	----------	-----	0.930 (0.831-1.041)	0.208	----------	-----	0.905 (0.803-1.020)	0.103	----------	-----
**TG**	0.998 (0.912-1.093)	0.969	----------	-----	1.113 (1.009-1.228)	0.033	1.032 (0.933-1.140)	0.542	1.044 (0.954-1.141)	0.348	----------	-----
**UA**	1.003 (1.001-1.004)	<0.001	1.002 (0.998-1.003)	0.244	1.003 (1.002-1.004)	<0.001	1.001 (0.999-1.002)	0.113	1.004 (1.002-1.005)	<0.001	1.002 (1.001-1.003)	0.046
**Creatinine**	1..020 (1.005-1.036)	0.005	0.823 (0.422-1.329)	0.201	1.033 (0.894-1.086)	0.165	----------	-----	1.157 (0.811-1.425)	0.148	----------	-----

Risk factors with p < 0.10 in unvariate adjusted simultaneously. Blanks indicate variables not adjusted in multivariate analyses.

**Table 6:** Univariate and multivariate logistic regression models for variables of male associated with calcified, non-calcified and mixed plaque (n = 566)

**V variable**	**Calcified plaque**				**Non-calcified plaque**				**Mixed plaque**			
	**Univariate**		**Multivariate**		**Univariate**		**Multivariate**		**Univariate**		**Multivariate**	
**Variable**	**OR (95%CI)**	**P**	**OR (95%CI)**	**P**	**OR (95%CI)**	**P**	**OR (95%CI)**	**P**	**OR (95%CI)**	**P**	**OR (95%CI)**	**P**
**Age**	1.069 (1.051-1.088)	<0.001	1.068 (1.049-1.087)	<0.001	1.018 (1.003-1.033)	0.016	1.024 (1.008-1.040)	0.003	1.048 (1.032-1.064)	<0.001	1.056 (1.038-1.075)	<0.001
**BMI**	0.979 (0.939-1.021)	0.315	----------	-----	1.009 (0.969-1.051)	0.664	----------	-----	0.986 (0.946-1.027)	0.500	----------	-----
**Smoking**	1.041 (0.745-1.456)	0.812	----------	-----	1.484 (1.058-2.082)	0.022	1.709 (1.194-2.447)	0.003	1.597 (1.140-2.237)	0.007	2.258 (1.548-3.294)	<0.001
**Family history of CAD**	0.771 (0.409-1.455)	0.422	----------	-----	1.409 (0.742-2.678)	0.295	----------	-----	1.488 (0.797-2.776)	0.212	----------	-----
**Hypertension**	1.745 (1.246-2.445)	0.001	1.522 (1.051-2.204)	0.026	1.291 (0.922-1.808)	0.137	----------	-----	1.533 (1.095-2.146)	0.013	1.411 (0.978-2.036)	0.066
**DM**	1.672 (1.086-2.575)	0.020	1.646 (1.027-2.637)	0.038	1.902 (1.209-2.991)	0.005	1.660 (1.043-2.642)	0.033	1.763 (1.144-2.716)	0.010	1.501 (0.942-2.393)	0.088
**LDL-C**	1.167 (0.948-1.436)	0.145	----------	-----	0.932 (0.757-1.146)	0.503	----------	-----	0.954 (0.775-1.173)	0.655	----------	-----
**HDL-C**	0.219 (0.094-0.344)	0.007	0.358 (0.169-0.547)	0.016	0.489 (0.271-0.882)	0.018	0.455 (0.248-0.832)	0.011	0.478 (0.261-0.874)	0.017	0.387 (0.200-0.749)	0.005
**TC**	1.058 (0.900-1.244)	0.496	----------	-----	0.970 (0.825-1.141)	0.717	----------	-----	0.851 (0.719-1.006)	0.059	0.946 (0.785-1.140)	0.561
**TG**	0.893 (0.787-1.013)	0.080	1.046 (0.925-1.182)	0.476	1.092 (0.967-1.233)	0.158	----------	-----	0.990 (0.890-1.102)	0.855	----------	-----
**UA**	0.999 (0.998-1.001)	0.601	----------	-----	1.001 (0.999-1.003)	0.232	----------	-----	1.001 (0.999-1.003)	0.498	----------	-----
**Creatinine**	1.061 (0.777-1.321)	0.543	----------	-----	1.980 (0.860-4.522)	0.126	----------	-----	0.631 (0.483-1.376)	0.194	----------	-----

Risk factors with p < 0.10 in unvariate adjusted simultaneously. Blanks indicate variables not adjusted in multivariate analyses.

**Table 7:** Univariate and multivariate logistic regression models for variables of female associated with calcified, non-calcified and mixed plaque (n = 550)

**V variable**	**Calcified plaque**				**Non-calcified plaque**				**Mixed plaque**			
	**Univariate**		**Multivariate**		**Univariate**		**Multivariate**		**Univariate**		**Multivariate**	
**Variable**	**OR (95%CI)**	**P**	**OR (95%CI)**	**P**	**OR (95%CI)**	**P**	**OR (95%CI)**	**P**	**OR (95%CI)**	**P**	**OR (95%CI)**	**P**
**Age**	1.101 (1.075-1.127)	<0.001	1.083 (1.056-1.111)	<0.001	1.057 (1.036-1.078)	<0.001	1.048 (1.026-1.070)	<0.001	1.101 (1.074-1.128)	<0.001	1.086 (1.058-1.115)	<0.001
**BMI**	1.041 (0.991-1.093)	0.110	----------	-----	1.073 (1.018-1.131)	0.008	1.047 (0.992-1.106)	0.098	1.038 (0.987-1.090)	0.145	----------	-----
**Smoking**	1.101 (0.557-2.177)	0.782	----------	-----	1.259 (0.670-2.366)	0.474	----------	-----	1.702 (0.877-3.302)	0.116	----------	-----
**Family history of CAD**	0.943 (0.494-1.800)	0.859	----------	-----	1.076 (0.599-1.933)	0.806	----------	-----	0.712 (0.346-1.464)	0.355	----------	-----
**Hypertension**	3.078 (2.101-4.508)	<0.001	2.216 (1.453-3.380)	<0.001	1.991 (1.411-2.810)	<0.001	1.506 (1.037-2. 188)	0.032	3.503 (2.340-5.244)	<0.001	2.720 (1.759-4.206)	<0.001
**DM**	4.093 (2.513-6.665)	<0.001	2.845 (1.666-4.858)	<0.001	1.868 (1.161-3.006)	0.010	1.287 (0.772-2.146)	0.333	3.383 (2.076-5.513)	<0.001	2.192 (1.281-3.750)	0.004
**LDL-C**	1.132 (0.930-1.379)	0.216	----------	-----	1.046 (0.870-1.257)	0.633	----------	-----	1.186 (0.967-1.455)	0.101	----------	-----
**HDL-C**	0.693 (0.404-1.188)	0.182	----------	-----	0.358 (0.206-0.622)	<0.001	0.486 (0.272-0.870)	0.015	0.965 (0.596-1.561)	0.884	----------	-----
**TC**	1.104 (0.926-1.315)	0.270	----------	-----	0.982 (0.834-1.156)	0.824	----------	-----	1.157 (0.965-1.388)	0.115	----------	-----
**TG**	1.202 (1.005-1.438)	0.044	1.111 (0.919-1.343)	0.277	1.111 (0.941-1.312)	0.215	----------	-----	1.112 (0.937-1.320)	0.224	----------	-----
**UA**	1.004 (1.002-1.007)	0.001	1.001 (0.998-1.434)	0.393	1.004 (1.001-1.006)	0.002	1.001 (0.998-1.004)	0.500	1.004 (1.002-1.007)	0.001	1.003 (0.001-1.006)	0.015
**Creatinine**	1.119 (0.866-1.288)	0.274	----------	-----	1.026 (0.687-1.537)	0.253	----------	-----	0.498 (0.173-2.051)	0.341	----------	-----

Risk factors with p < 0.10 in unvariate adjusted simultaneously. Blanks indicate variables not adjusted in multivariate analyses.

## Discussion

In this study of individuals with suspected CAD using 256-detector-row CCTA, we analysed the association of UA with coronary atherosclerosis. We found the following findings. 1) Overall and in women, the prevalence of coronary atherosclerosis, severe stenosis, and triple-vessel/left main artery lesions of plaques were significantly increased with increasing quartiles of UA. UA was significantly associated with double-vessel lesions in women. 2) UA was significantly negatively correlated with the proportion of a CACS of 0, but was positively correlated with the proportion of a CACS >10 overall and in women. The incidence of mixed plaques was significantly increased only in women with elevated UA levels. 3) After adjustment, only UA was a significant predictor of significant stenosis, multivessel disease, and mixed plaques in women. UA was not significantly associated with coronary atherosclerosis in men.

Coronary artery angiography (CAG) is the gold standard for diagnosing CAD, but CAG cannot determine the characteristics of plaques. CAG would be difficult to use as a general screening tool in China because of its highly invasive nature, radiation exposure, and cost. The technique of 256-detector-row CCTA with a shorter scanning time can improve image quality and it has a minimal radiation dose [18]. Use of CCTA in a general routine health evaluation is not discouraged [19]. Therefore, healthy subjects were not included in our study. We enrolled those patients with suspected CAD who needed to have a CCTA examination while undergoing a clinician’s assessment. CCTA is a screening tool for assessing individuals of suspected CAD, and is the most feasible and effective method to reflect widespread epidemiology and characteristics of early CAD in China.

Many studies have assessed the association of UA with CAD and clinical outcome, but the results were still controversial. Kocaman et al. found that UA was an independent predictor of CAD in individuals undergoing CAG [20]. However, the Framingham studies showed that UA was not an independent predictor of CAD and cardiovascular outcomes [21], and similar results were found in the ARIC study [22] and a study by Strask et al. [23]. Carotid intima-media thickness (C-IMT) measured by ultrasonography is widely used as a surrogate marker for atherosclerotic disease and directly associated with increased risk of cardiovascular disease. The higher UA levels were associated with higher C-IMT independent from hypertension, UA levels were independently associated with C-IMT [24,25]. But a study shown UA was not significantly associated with C-IMT, UA was associated with Internal carotid artery resistive index in hypertensive women, suggesting that there might gender-related differences in the relationship between UA and vascular damage [26]. In our study, although the prevalence and degree of plaques were significantly increased with UA overall, UA was not a significant independent predictor. Several studies have shown conflicting results on the association between UA and CAD in men and women. Some studies have demonstrated that UA was an independent risk factor for CAD in both sexes [13,14]. A high UA level was found to be an independent predictor for cardiovascular mortality in men [27]. In the LIFE study, the significant association of UA with CAD was found only in women [12]. Cardiovascular death was increased by 1.77 times in men and 3.0 times in women in the upper UA quartile in the NHANES I study [28]. A previous study reported that UA levels only in women were associated with CAD [29]. Our study supported the opinion that UA was an independent predictor for the prevalence and severity of early CAD only in women.

Previous reports have proven the highly predictive value of the CACS on cardiovascular outcomes [30,31]. Some studies have shown a significant relationship between UA and the CACS [32-34], and a recent study showed that UA was an independent factor predictive of the CACS [34]. However, some studies did not support these previous findings [7,35]. In our study, we found that UA was significantly associated with the CACS, but UA was not an independent risk factor for a high CACS overall, in men and women. The relationship between UA and CACS, and the concrete mechanisms are still unclear. Few studies have focused on the role of UA in characterization of plaques as shown by CCTA. One study showed that UA was only significantly associated with calcified plaques [36]. In our study, only UA was an independent predictor for mixed plaques overall and in women. The relationship between UA and mixed plaques was likely caused by the female subgroup. Many studies have indicated that mixed plaques might be the biggest risk of plaque rupture, causing acute coronary events [37,38]. This may be a rational explanation for why high UA level was associated with an increase in the prevalence of mixed plaques in women, which causes an increase in the incidence of adverse cardiovascular outcomes. However, there are too few studies on the relationship between UA and plaque characteristics, and further studies are required.

UA is a general antioxidant in the body, and a high UA level is suggestive of oxidative stress, endothelial dysfunction, and slow coronary artery flow [39,40]. UA promotes vascular smooth muscle proliferation, and upregulates the expression of monocyte chemoattractant protein-1 and platelet-derived growth factor [41]. Endothelial dysfunction is an important step in the development of atherosclerosis. A recent study showed a relationship between UA and endothelial dysfunction of coronary microvasculature only in women [42], which may be a possible explanation for the significant association between UA and CAD in women. The underlying mechanisms behind the significant relationship between UA and CAD in women but not men remain enigmatic. Our study suggests that UA plays an important role in coronary atherosclerosis in women.

The incidence of cardiovascular events is still high after controlling for traditional risk factors. Our study suggests that UA should be considered as an additional risk factor beyond traditional factors for CAD in women. However, a randomized study still needs to be performed to determine the effect of UA-lowering therapies on cardiovascular prevention. In the GREACE study, decreased UA levels by atorvastatin were independently correlated with a reduced risk of cardiovascular outcomes [43]. The LIFE study showed an association between UA and cardiovascular events in hypertensive women, the unique results may be due in part to the specific feature of reduction of UA by losartan, but it did not mean that the beneficial effects observed with losartan were contributed to decreasing UA levels [12]. The relationship between low UA level and cardiovascular benefits is still uncertain. Therefore, further research should be performed to assess the direct relationship between decreasing UA level and cardiovascular benefits.

There were several limitations to our study. All individuals were suspected of having CAD, we mainly want to explore the relationship between UA and early CAD in the study. Patients with known CAD may implement lifestyle modifications and drugs intervention to some extent, the real relationship between UA and CAD may be influenced by those confounding factors, So, we exclude the patients who had known CAD. There was a possible selection bias and this could restrict generalizability of our results to similar care settings. Unmeasured confounders may have affected our results. In our study, almost 90% of women were post-menopausal and few women received oestrogen-replacement therapy. Therefore, we did not analyse the potential effects of oestrogen on the relationship between UA and CAD. In cross-sectional analysis, we did not find a longitudinal relationship between UA and cardiovascular outcomes. In spite of these limitations, the strong association of UA with early CAD in women had an important clinical significance in prevention and treatment of CAD. Longitudinal studies are required to confirm the association of UA with CAD and cardiovascular outcome.

## Conclusion

UA is a significant predictor of significant stenosis, multivessel disease, and mixed plaques in women. Importantly, our study suggests that UA level may play an important role in the occurrence and development of coronary atherosclerosis in women but not men.

## Abbreviations

UA: Uric acid; CAD: Coronary artery disease; CCTA: Coronary computed tomography angiography; CACS: Calcium score; BMI: Body mass index; TC: Total cholesterol; TG: Triglyceride; HDL-C: High-density lipoprotein cholesterol; LDL-C: Low-density lipoprotein cholesterol; CAG: Coronary artery angiography.

## Competing interests

The authors declare that they have no competing interest.

## Authors’ contribution

SYJ participated in the design of the study, collected and organized datas, analysed images, performed the statistical analysis and drafted the manuscript. YX collected, organized and analysed datas. ZY, GS and LH collected and organized datas. LT and XK analysed images. CL and WCW collected datas. QGX conceived of the study, and participated in its design and coordination and helped to draft the manuscript. All authors read and approved the final manuscript.

## Pre-publication history

The pre-publication history for this paper can be accessed here:

http://www.biomedcentral.com/1471-2261/14/101/prepub
